# *TUG1* supports mouse two-cell-stage development and ZGA-associated transcription through MAPK8 signaling

**DOI:** 10.1007/s00018-026-06374-z

**Published:** 2026-08-11

**Authors:** Jianwu Wang, Guang Yang, Qingbo Yang, Hongshuang Xie, Yiwei Zhang, Jiaqiang Wang

**Affiliations:** Key Laboratory of Animal Cellular and Genetics Engineering of Heilongjiang Province, College of Life Science, Northeast Agricultural University, Harbin, 150030 P. R. China

**Keywords:** Long non-coding RNAs, Zygotic genome activation, Mouse embryos, Two-cell arrest

## Abstract

Cancer cells possess unlimited proliferative potential, and the high expression of lncRNAs widely promotes cancer progression. Mammalian early embryos share similar characteristics, where numerous endogenous retroviruses become transcriptionally active during zygotic genome activation (ZGA). However, whether cancer-promoting lncRNAs can also facilitate early embryonic development remains unclear. In this study, we demonstrated that *TUG1*, which is highly expressed in tumors, regulates early embryonic development in mice. *TUG1* deficiency results in the arrest of mouse embryos at the two-cell stage, accompanied by disruption of the MAPK signaling pathway, and consequently leads to the failure of ZGA. Mechanistically, we uncover a *TUG1*-hnRNPA1 ribonucleoprotein complex that actively licenses ZGA by promoting the transcription of MAPK8, thereby activating the essential MAPK signaling pathway. This study transforms our understanding of *TUG1* from a cancer-specific effector to a critical regulator of the ZGA, raising the possibility that related regulatory principles may operate in other biological contexts.

**Supplementary Information:**

The online version contains supplementary material available at https://doi.org/10.1007/s00018-026-06374-z.

## Introduction

Long non-coding RNAs (lncRNAs) are a class of non-coding RNA molecules with lengths exceeding 200 nucleotides, transcribed by RNA polymerases I, II, and III [1]. They often exhibit marked cell-type specificity and play crucial regulatory roles in processes such as cell differentiation, organismal development, and diverse physiological functions [2–4]. Recent discoveries of competitive endogenous RNAs (ceRNAs) and circular RNAs (circRNAs) have further expanded the known complexity of lncRNA-mediated regulatory networks [5, 6]. Studies have shown that lncRNAs can modulate gene expression at both transcriptional and post-transcriptional levels [7, 8]. Accumulating evidence indicates that dysregulation of lncRNA expression is implicated in the pathogenesis of various cancers [9, 10]. Furthermore, lncRNAs are actively involved in embryonic development. For instance, *LincGET* and *GAS5* regulate early embryonic cell fate in mice by promoting differentiation toward the inner cell mass (ICM) and trophectoderm (TE), respectively [11, 12]. In pigs, the SINE-associated lncRNA *SAWPA* and the enhancer-like *lncFKBPL* influence zygotic genome activation [13, 14]. Therefore, investigating the regulatory mechanisms of lncRNAs could not only help identify new therapeutic targets for cancer, but also enhance the potential for treating reproductive diseases.

During mammalian preimplantation embryonic development, a series of critical events take place, including zygotic genome activation (ZGA), embryonic polarization and compaction, and the first lineage segregation [15–17]. As the first transcriptional milestone after fertilization, ZGA represents a crucial turning point that initiates the embryonic developmental program and facilitates the transition from maternal to zygotic control of gene expression [18, 19]. To date, numerous transcription factors associated with ZGA—such as HSP70, Zscan4 and members of the OBOX family—have been identified based on their expression patterns and functional roles [20–22]. Additionally, the activation of MuERV-L-associated long non-coding RNAs (lncRNAs) serves as a key mechanistic link between fertilization-induced epigenetic reprogramming and the activation of the embryonic gene expression program [23]. Consequently, understanding how lncRNAs regulate ZGA remains a major focus of ongoing research.

Taurine-upregulated gene 1 (*TUG1*) was initially identified in retinal cells of newborn mice, with its deficiency leading to impaired retinal development [24]. *TUG1* is closely associated with cancer cell proliferation, invasion, migration, metastasis, and apoptosis. As research on its mechanisms in cancer cells continues to deepen, it shows promise as a potential therapeutic target. In renal cancer cells, *TUG1* promotes proliferation and migration by regulating the miR-9/YAP pathway [25]. In laryngeal cancer cells, highly expressed *TUG1* activates the RhoA/Rho-associated coiled-coil kinase MMPs signaling pathway by inhibiting miR-145-5p, thereby promoting the migration and invasion of cancer cells [26]. Furthermore, studies in various cancer cells have shown that *TUG1* interacts with RPA and DHX9 to activate the ATR-CHK1 signaling pathway, eliminating R-loop accumulation in cancer cells [27]. However, research on *TUG1* has primarily focused on cancer, and its role in early embryonic development remains largely unknown.

In this study, we identified pro-oncogenic lncRNAs that are specifically and highly expressed at the mouse 2-cell stage. Depletion of *TUG1* resulted in embryonic arrest at the two-cell stage and was associated with disruption of the MAPK signaling pathway. Further investigation revealed that *TUG1* forms an RNA-protein complex by binding to hnRNPA1, which in turn enhances the transcription of MAPK8, thereby facilitating the proper initiation of ZGA. Collectively, these findings indicate that *TUG1* plays an essential role in ensuring the successful execution of ZGA in mouse preimplantation embryos.

## Materials and methods

### Embryo collection

All experiments were performed in accordance with ARRIVE guidelines and regulations. To obtain preimplantation embryos, 7‑week‑old ICR female mice were intraperitoneally injected with 10 IU of pregnant mare serum gonadotrophin (PMSG), followed by an intraperitoneal injection of 10 IU of human chorionic gonadotrophin (hCG) 48 h later. The females were then housed with 7‑week‑old ICR male mice. Vaginal plugs were checked the next morning, and females with a vaginal plug were designated as embryonic day 0.5. Embryos were collected in M2 medium and cultured in pre‑equilibrated KSOM medium. Zygotes were collected 20 h after hCG administration.

### Primer and probe design

All primers and probes were designed using PrimerPremier5 and synthesized from Comate Bioscience. Primers are shown in Supplementary Table 1.

### Antibodies

The following antibodies were used for Western blot, Immunofluorescence and co-IP assay: anti-MAPK8 (Proteintech, 24164-1-AP), anti-Phospho-MAPK8 (Tyr185) (Proteintech, 80024-1-RR), anti-BrdU (#ab1893; Abcam), anti-β-Actin (Abcam, ab197345), anti-hnRNPA1 (Proteintech, 11176-1-AP), anti-HA Tag (Abcam, ab1424). Secondary antibodies: Donkey Anti-Mouse IgG H&L (Alexa Fluor 488) (abcam, #ab150105), HRP-conjugated goat anti-Rabbit IgG polyclonal secondary antibody (Invitrogen, A27036), and HRP-conjugated rabbit anti-Mouse IgG polyclonal secondary antibody (Invitrogen, 616520).

### Microinjection

For *TUG1* knockdown and mRNA overexpression, RNA (siRNA, 20 µM; mRNA, 150 ng/µL) was loaded into microneedles using a FemtoJet microinjector (Eppendorf, Germany). Equal volumes were injected per embryo under the following conditions: injection pressure 50 hPa, compensation pressure 20 hPa, and injection duration 0.3 s. Each embryo received approximately 5–10 pL of solution. Post-injection, embryos were transferred to an incubator for cultivation.

### Culture cells and transfection

293T cells were cultured in a fibronectin-coated dish in a fibroblast culture medium consisting of 15% fetal bovine serum (BI, 04-002-1 A), 1% non-essential amino acids (Invitrogen, 11140050), 1% glutamax (Gibco, 35050061), 1% penicillin–streptomycin–glutamine (Gibco, 10378016), and Dulbecco’s modified Eagle’s medium (Gibco, 11965092) at 37 °C in a 5% CO2 incubator. The culture medium was changed every day. 293T cells were passaged every 2–3 days and then digested to single cells by 0.05% trypsin/EDTA (Gibco, 25300062). For cell transfection, 293T were passaged and seeded at a density of 10,000–15,000 cells/cm^2^. After 2 days (60%–70% confluence), plasmid DNA with or without LNA targeting *TUG1* was transferred into cells using LipofectamineTM 3000 transfection reagent (Gibco, L3000015) according to the manufacturer’s instructions. 293T were collected for further analysis after 48 h.

### RNA-FISH

Perform the assay using a Type G in situ hybridization detection kit (Focofish, D-0010). Embryos underwent fixation in 4% paraformaldehyde. Pre-hybridization involved sequential treatments: Solution A (37 °C, 15 min), Solution B (37 °C, 15 min), three PBS washes, Solution C (37 °C, 20 min), three additional PBS washes, 3% H₂O₂ incubation (RT, 15–20 min), and final fixation in 4% PFA (RT, 10 min) followed by three PBS rinses. After complete air-drying, Cy3-UTP-labeled probes (Jenerbio) were denatured (85 °C, 4 min) in Hybridization Buffer and equilibrated (37 °C, 2 min). Hybridization was performed with 50 µL probe mixture (37 °C, 18 h). Post-hybridization washes included: 1× Washing Buffer (37 °C, 10 min) and fresh buffer (RT, 5 min). Nuclei were counterstained with DAPI-antifade solution before mounting and imaging.

### Data analysis

Statistical analyses [mean ± standard error of the mean (SEM)] for differential gene expression, differential fluorescence intensity, and differential abundance on gels were performed using GraphPad Prism 8. Levels of significance were calculated with Student’s *t*-tests. Isoform abundance on SDS-PAGE gels or agarose gels was measured in Fiji/ImageJ.

## Results

### *TUG1* is highly expressed in mouse embryos at the two-cell stage

**Fig. 1:**
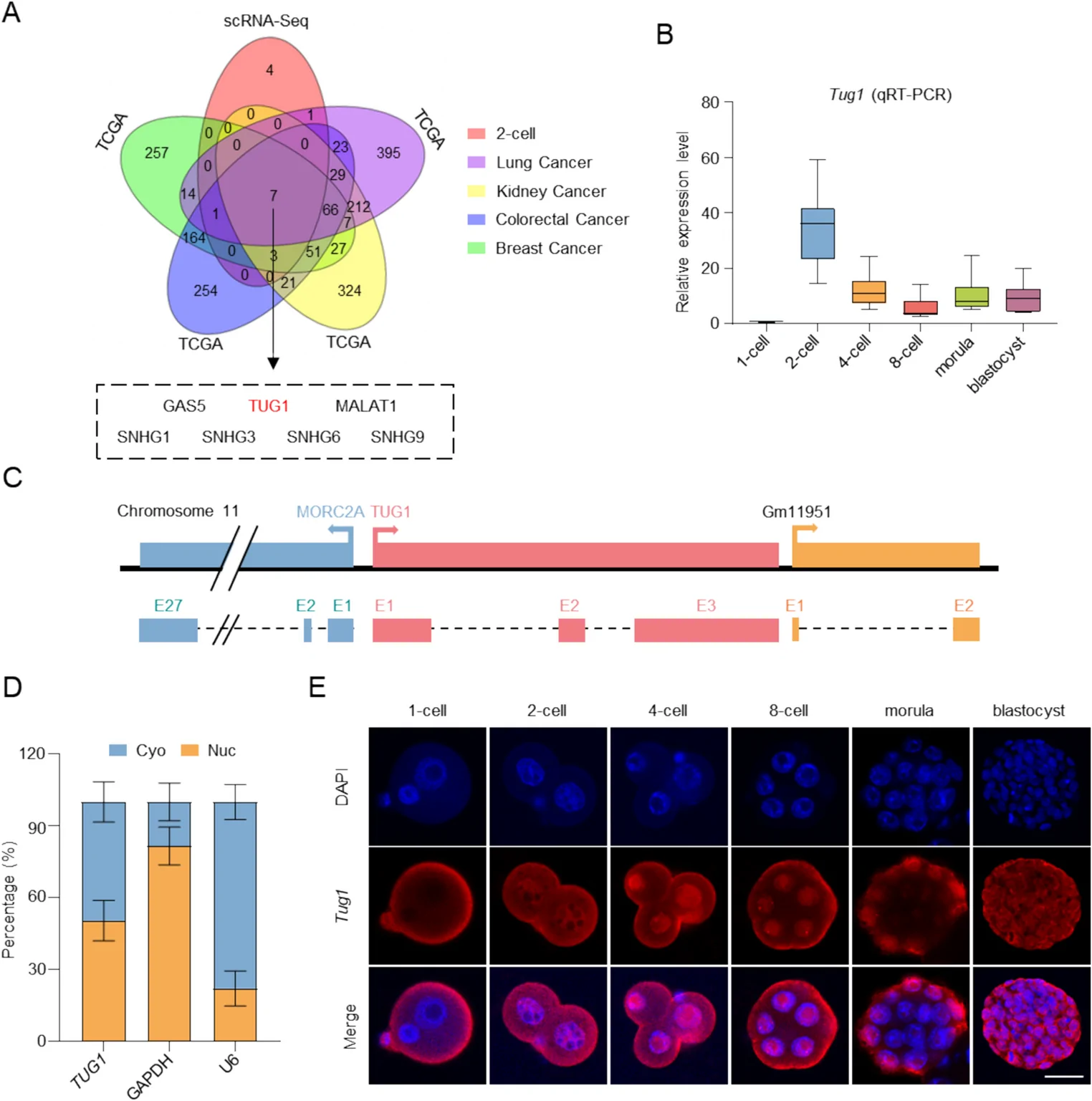
*TUG1* genomic locus, expression pattern, and cellular localization (**A**) Venn diagram illustrating seven lncRNAs with high expression potential across different cancer cells and mouse 2-cell stage embryos, and the data was obtained from TCGA and GEO (GSE45719) (**B**) Quantitative PCR analysis of relative *TUG1* expression in embryos at different developmental stages. Experiments were performed with three independent biological replicates, using Hprt as the reference gene. Box plots show medians (center lines) and interquartile ranges (box edges) (**C**) Gene locus of *TUG1*. It is located downstream of MORC2A and consists of three exons (**D**) Subcellular localization analysis of *TUG1* by RNA fractionation and RT-qPCR analysis. The results show that *TUG1* exhibits both cytoplasmic and nuclear distribution. The error bars represent SEM. Cyt, cytoplasm; Nuc, nucleoplasm. Gapdh and U6 act as cyt and nuc control, respectively. About 500 two-cell embryos were used for each experiment. *n* = 3 biologically independent experiments (**E**) RNA-FISH detection of TUG1 in 1-cell to blastocyst stage embryos. 1-cell (n = 7), 2-cell (n = 7), 4-cell (n = 6), 8-cell (n = 6), morula (n = 7), blastocyst (n = 7). Scale bar: 50 μm

To identify potential lncRNAs in cancer cells and early embryos, we conducted a comprehensive analysis of multiple RNA-sequencing datasets, including lncRNA expression profiles from TCGA along with scRNA-seq data associated with mouse 2-cell stage embryos [28]. Subsequently, lncRNAs with a fold-change ≥ 2 were carefully selected for Venn analysis, ultimately identifying seven promising candidates: *GAS5, TUG1, MALAT1, SHNG1, SHNG3, SHNG6*, and *SHNG9*, all of which showed significant expression levels (Fig. 1A). Through comparative analysis of RPKM values across different embryonic developmental stages, we found that the expression dynamics of *TUG1* were the most pronounced among the candidates, only *TUG1* exhibited specific high expression at the 2-cell stage (Supplementary Fig. S1A-G). We also collected embryos at various stages and verified the expression pattern of *TUG1* in preimplantation embryos using qPCR, which was consistent with the RNA-seq results (Fig. 1B). Its expression peaks at the two-cell stage, continuously decreases from the two-cell to eight-cell stage, but shows a slight increase again at the blastocyst stage (Fig. 1B). Therefore, we selected TUG1 as the focus of this study. We first investigated the genomic location of *TUG1*. Using the BLAT tool from UCSC, we determined that *TUG1* is located on chromosome 11 and consists of three exons (Fig. 1C).

Next, we performed subcellular localization analysis. By separating two-cell embryos into cytoplasmic and nuclear fractions, followed by RNA extraction and SYBR Green-based quantitative PCR (qPCR), we examined the localization of Gapdh, U6, and *TUG1*. We found that *TUG1* is present in both the cytoplasm and nucleus (Fig. 1D). To further clarify the expression pattern of *TUG1*, we conducted RNA-FISH on preimplantation embryos. *TUG1* was distributed in both the cytoplasm and nucleus of early embryonic blastomeres (Fig. 1E). Interestingly, *TUG1* exhibits a dynamic spatial distribution pattern. At the one-cell stage, *TUG1* localizes primarily to the subcortical region. By the two-cell stage, it begins to translocate into the nucleus. From the four-cell to eight-cell stages, it is predominantly located in both subcortical regions and the nucleolus. During the morula stage, *TUG1* is mainly enriched in outer cells, while at the blastocyst stage, its expression becomes restricted to the TE, with no detectable signal in the ICM. This dynamic localization pattern may reflect a distinct regulatory mechanism of *TUG1*. Furthermore, the stage-specific high expression of *TUG1* in mouse two-cell embryos suggests a potential functional role at this stage.

### *TUG1* knockdown results in embryonic arrest at the two-cell stage

**Fig. 2:**
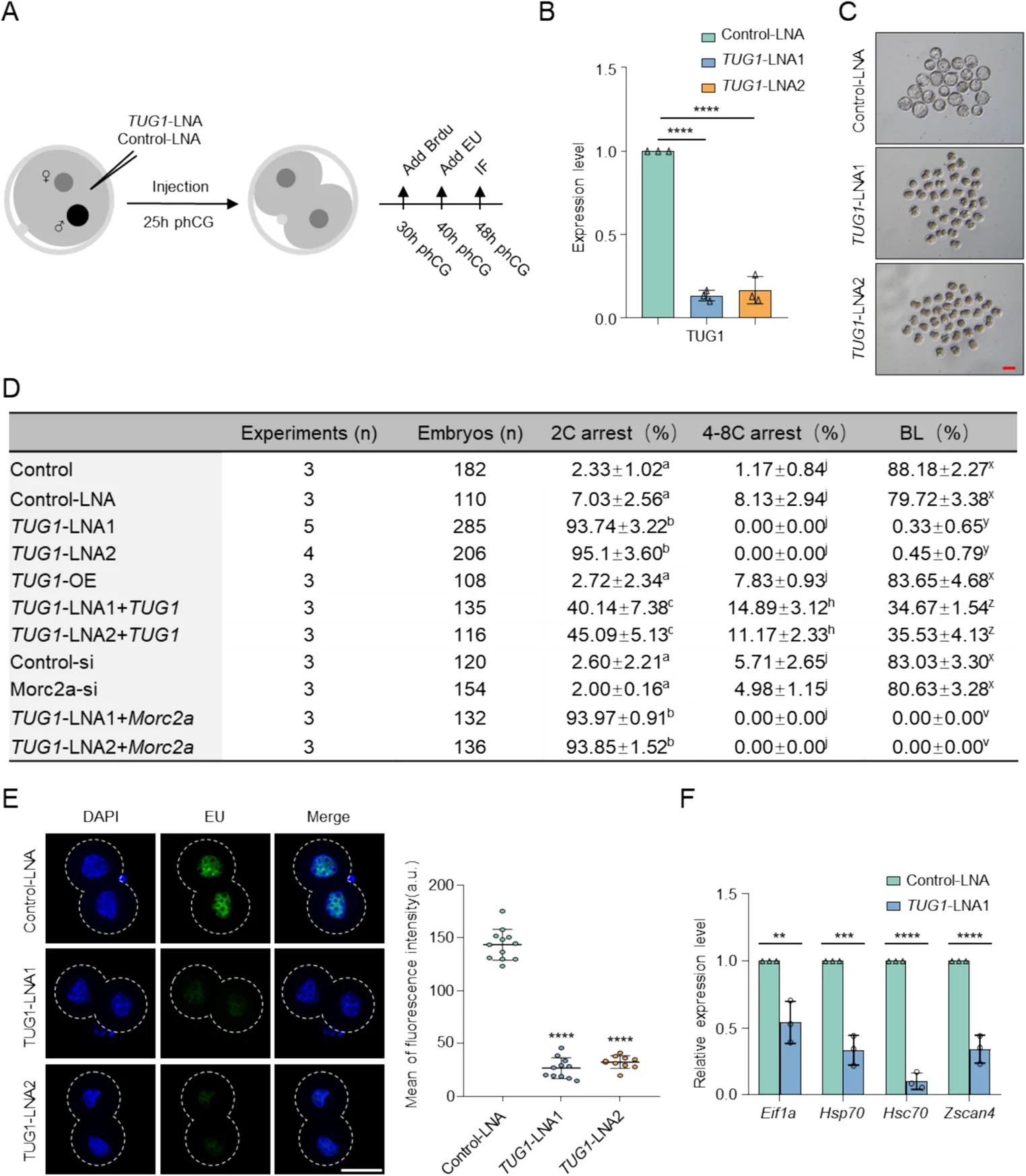
*TUG1* knockdown results in embryonic arrest at the two‑cell stage and impairs ZGA in mice (**A**) Experimental scheme to analyze the effects of *TUG1* depletion on embryonic development. phCG, post-human chorionic gonadotropin injection; LNA, locked nucleic acid; IF, immunofluorescence. LNA was injected at phCG 25 h. For BrdU staining, BrdU was added at phCG 30 h. For EU staining, EU was added at phCG 40 h. IF, including detection of BrdU and EU, was performed at phCG 48 h (**B**) Locked nucleic acid (LNA) efficiently mediated *TUG1* knockdown. Design two LNA-modified siRNA fragments. LNA was injected at phCG 25 h, and embryos were collected at phCG 48 h at late two-cell stage for RT-qPCR analysis. The control group represents the LNA-modified siRNA control fragments. About 100 embryos were used, and n = 3 biologically independent experiments (**C**) Embryonic morphology at preimplantation stages following TUG1 knockdown and overexpression. Scale bar: 100 μm (**D**) Embryonic development after microinjection. Different letters in same column indicate significant difference (*p* < 0.001) (**E**) EU staining after TUG1 knockdown. EU was added to the culture medium at phCG 40 h, and EU signals were detected at phCG 48 h. Scale bar, 50 μm. Quantification of fluorescence intensity from at least 8 representative pictures. n = 3 biologically independent experiments (**F**) *TUG1* knockdown reduces expression of ZGA-related genes. Embryos injected with LNA were collected at phCG 48 h at the late two-cell stage for qPCR analysis. The error bars represent SEM. About 100 embryos were used, and three experimental replicates were performed. For all panels, error bars represent SEM. Significance was determined by two-tailed t-test, *p ≤ 0.05; **p ≤ 0.01; ***p ≤ 0.001; ****p ≤ 0.0001

To investigate the role of *TUG1* in early embryonic development, we performed knockdown experiments using locked nucleic acid (LNA)-modified siRNA (Fig. 2A). Control LNA or *TUG1*-targeting LNA (*TUG1*-LNA) was injected into zygotes. Two LNA sequences (LNA1 and LNA2) were screened, both achieving nearly 90% knockdown efficiency (Fig. 2B). We then evaluated embryonic development and observed that depletion of *TUG1* (*TUG1*-LNA1 and *TUG1*-LNA2) resulted in developmental arrest of mouse embryos at the two-cell stage (Fig. 2C, Supplementary Fig. S2A). Embryonic development following injection of the corresponding RNA fragments was assessed, and statistical analysis confirmed that embryos injected with *TUG1*-LNA1 or *TUG1*-LNA2 arrested at the two-cell stage (Fig. 2D). Meanwhile, overexpression of *TUG1* did not affect embryonic development (Fig. 2D). Additionally, a rescue experiment was conducted on embryos arrested at the two-cell stage. Co-injection of full-length *TUG1* containing mutations at the LNA target sites restored development to the blastocyst stage in approximately 40% of the embryos, significantly rescuing the arrest phenotype (Fig. 2D).

### *TUG1* knockdown impairs major ZGA

Mouse ZGA comprises two waves: the minor ZGA at the one-cell stage and the major ZGA at the two-cell stage [29–31]. The proper occurrence of ZGA, particularly the major ZGA, is essential for normal early embryonic development and the precise determination of cell fate [32]. Given that ZGA is a period of massive transcription, to determine whether interfering with *TUG1* affects ZGA, we first performed BrdU staining. The results showed no significant difference in BrdU fluorescence intensity between the *TUG1*-knockdown group and the control group, indicating that *TUG1* interference did not affect DNA replication (Supplementary Fig. S1B). We then used 5-ethylnyl uridine (EU) labeling to detect the level of nascent transcripts and assess whether *TUG1* interference affects the initiation of major ZGA at the two-cell stage. The results showed a significant difference in EU signals between the control-LNA group and the *TUG1*-LNA group, with the fluorescence intensity in the knockdown group being much lower than that in the control group (Fig. 2E). We also analyzed the expression changes of ZGA-initiating genes *Eif1a*, *Hsp70*, *Hsc70*, and *Zscan4*. Their expression levels were significantly reduced after *TUG1* knockdown (Fig. 2F). These results demonstrate that interference with *TUG1* impairs major ZGA.

### *TUG1* is a non-panc lncRNA

**Fig. 3:**
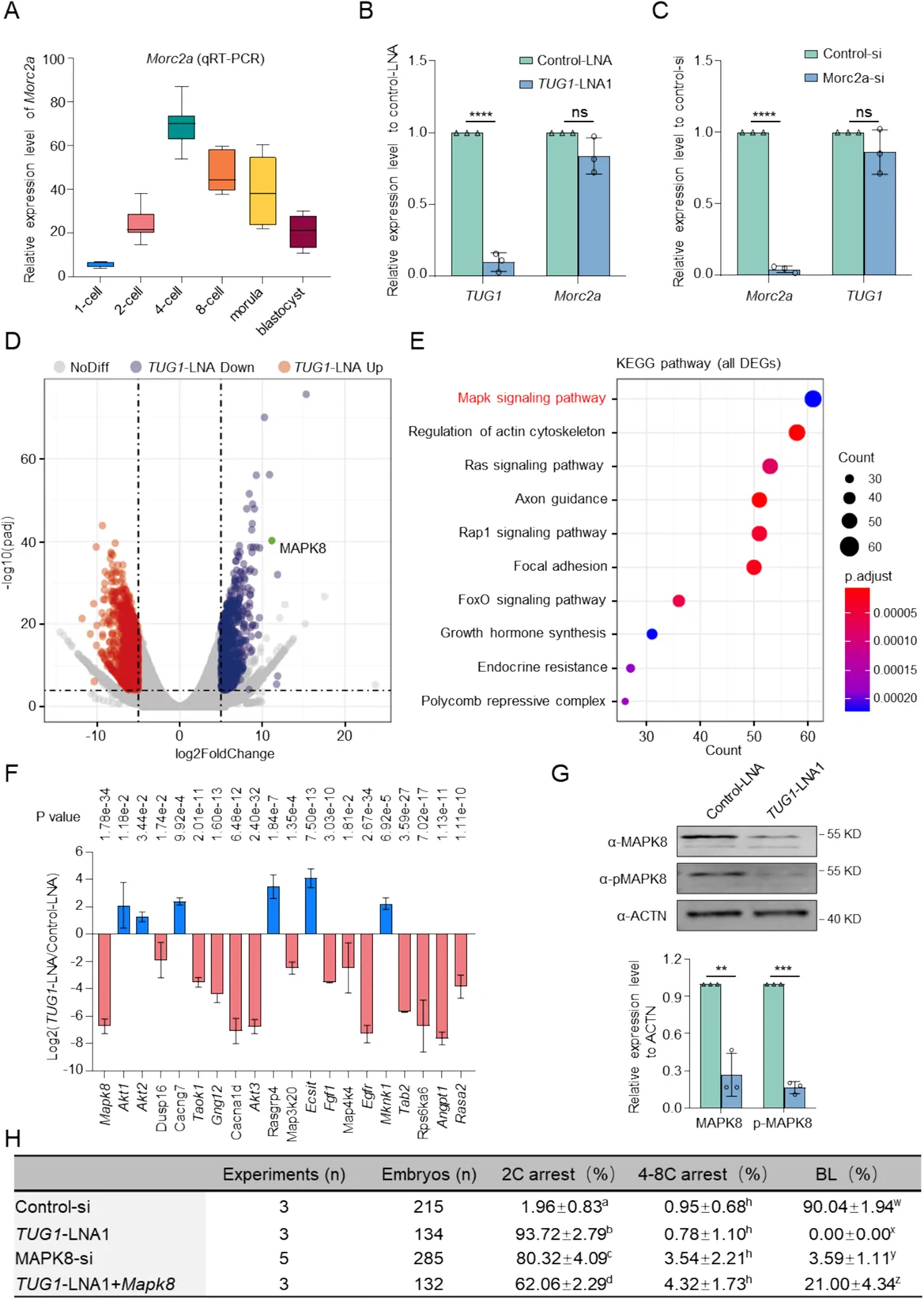
Knockdown of*TUG1*impairs the MAPK signaling pathway and consequently downregulates MAPK8 expression (**A**) Quantitative PCR analysis of relative *Morc2a* expression in embryos at different developmental stages. *n* = 3 biologically independent experiments, using* Hprt* as the reference gene. Box plots show medians (center lines) and interquartile ranges (box edges) (**B**) Following injection of Control-LNA and *TUG*1-LNA1, expression analysis of *TUG1* and *Morc2a* was performed in the control group. About 50 embryos were used, and *n* = 3 biologically independent experiments (**C**) Following injection of Control-si and Morc2a-si, expression analysis of *TUG1* and *Morc2a* was performed in the control group. About 50 embryos were used, and *n* = 3 biologically independent experiments (**D**) The volcano plot illustrates the differential gene expression analysis of the RNA-seq data. Compared to the Control-LNA group, 956 genes were upregulated, and 692 genes were downregulated in *TUG1*-depleted embryos (**E**) KEGG pathway analysis of DEGs showed that the MAPK signaling pathway is mainly affected by *TUG1* depletion (**F**) The expression of key factors in the MAPK8-MAPK signaling pathways was significantly affected by *TUG1* depletion. Embryos injected with LNA were collected at phCG 48 h at late two-cell stage for qPCR analysis. About 100 embryos were used for each group, and n = 3 biologically independent experiments (**G**) Western blot and grayscale analysis indicate that the protein and phosphorylation levels of MAPK and pMAPK, key kinases in the MAPK signaling pathway, decreased in* TUG1*-depleted 2-cell. Embryos injected with Control-LNA and *TUG1*-LNA1 were collected at phCG 48 h at the 2-cell stage for western blot analysis, and about 200 (**H**) Embryonic development after *TUG1*-LNA1 and Mapk8-si microinjection. 2 C, 2-cell stage; 4–8 C, 4-to 8-cell stage; BL, blastocyst stage; si, siRNA. siRNA concentration for both control and knockdown groups was 150 ng/µL. Different letters within the same column indicate statistically significant differences (P < 0.001). For all panels, error bars represent SEM. Significance was determined by two-tailed t-test, *p ≤ 0.05; **p ≤ 0.01; ***p ≤ 0.001; ****p ≤ 0.0001, ns not significant

Many lncRNAs exert cis-regulatory effects through adjacent genes and belong to the category of panc-lncRNAs [13]. Since MORC2A is located upstream of *TUG1*, we initially hypothesized that *TUG1* might be a panc-lncRNA and could have a regulatory relationship with its neighboring gene MORC2A. To test this, we first analyzed the expression level of *Morc2a* during different embryonic stages. The results showed that *Morc2a* mRNA levels exhibited dynamic changes in early mouse embryos, peaking at the four-cell stage and gradually declining from the four-cell to blastocyst stage, which differed from the expression pattern of *TUG1* (Fig. 3A). Next, we injected knockdown fragments targeting either *TUG1* or *Morc2a* at the one-cell stage. qPCR results revealed that knockdown of *TUG1* did not affect *Morc2a* expression, and similarly, *Morc2a* knockdown had no effect on *TUG1* expression (Fig. 3A and B). Further analysis of embryonic development after *Morc2a* knockdown showed no impact on early development, with blastocyst rates comparable to the control group (Fig. 2D). We also performed co-injection of *TUG1*-LNA and full-length *Morc2a* mRNA, which demonstrated that *Morc2a* could not rescue the two-cell arrest phenotype (Fig. 2D). In summary, these results indicate that there is no regulatory relationship between *TUG1* and *Morc2a*, and that *TUG1* is a non-panc lncRNA.

### *TUG1* knockdown inhibits the MAPK signaling pathway

To elucidate the mechanism underlying the embryonic arrest at the two-cell stage induced by *TUG1* depletion, we compared gene expression between control-LNA and *TUG1*-LNA groups using low-input RNA-Seq. Overall, compared with the control-LNA group, the *TUG1*-LNA group showed 1,648 differentially expressed genes (*p* ≤ 0.05, RPKM ≥ 1, log₂FoldChange > 1), of which 692 were upregulated and 965 were downregulated (Fig. 3D). Among the downregulated genes, 17.4% were related to major ZGA and 6.6% to maternal genes, whereas no alterations were observed in minor ZGA‑associated genes. This finding is consistent with the EU staining results, suggesting that the 2‑cell arrest of *TUG1*‑deficient embryos results from compromised major ZGA(Supplementary Fig. S3A and 3B). KEGG pathway analysis of differentially expressed genes revealed significant enrichment of the MAPK signaling pathway following *TUG1* depletion (Fig. 3E). We validated the expression changes of key genes in the MAPK pathway by qPCR. In *TUG1*-depleted embryos, the expression of *Mapk8* was reduced by more than 6-fold (Fig. 3F). Furthermore, western blot analysis revealed a significant decrease in phosphorylated MAPK8 levels as well as a reduction in total MAPK8 protein in *TUG1*-knockdown embryos (Fig. 3G). The embryos injected with MAPK8-si were affected by blastocyst rate, and embryos arrest and 2-cell stage. We then co-injected *TUG1*-LNA embryos with overexpression of *Mapk8* mRNA; overexpressed *Mapk8* mRNA rescued embryonic development arrest compared to the *TUG1*-LNA group (Fig. 3H). These results indicate that the MAPK signaling pathway plays a crucial role in early mouse embryonic development.

### *TUG1* interacts with hnRNPA1

**Fig. 4:**
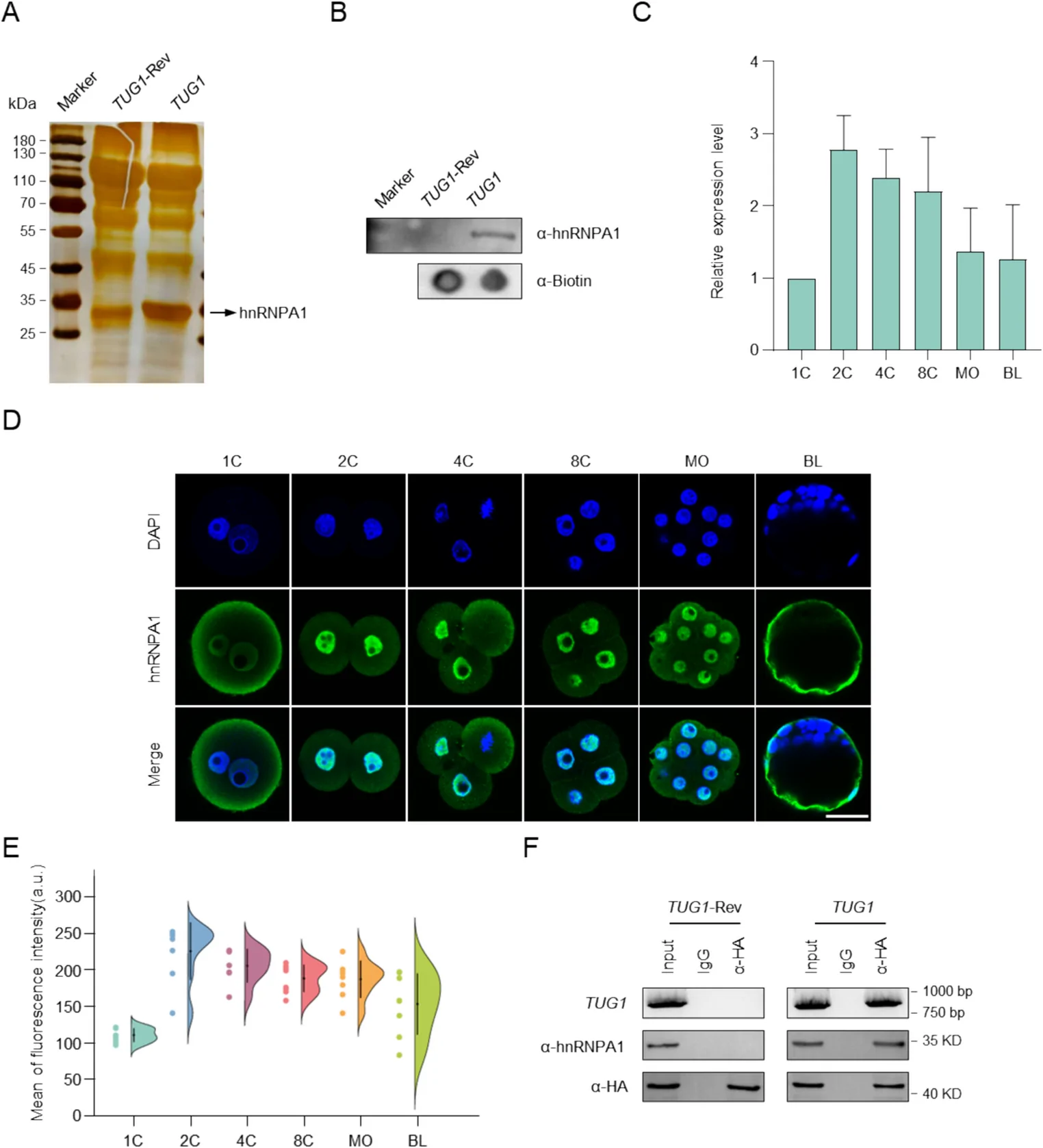
*TUG1 *binds to hnRNPA1 (**A**)*TUG1* interacts with hnRNPA1 in vitro. The samples of RNA pull-down experiment were subjected to SDS-PAGE gel electrophoresis, and then the binding protein of *TUG1* and *TUG1*-rev was analyzed by silver staining and MS. Pull-down assay for mass spectrometry analysis was performed with 6000 2-cell stage embryos. *TUG1*‑rev, the reverse 1500‑bp sequence of *TUG1* (**B**) Mass spectrometry results of hnRNPA1 were confirmed by western blot following RNA pull-down assay; α-, anti-. For each pull-down WB assay, about 1,000 two-cell embryos were used and *n* = 3 biologically independent experiments (**C**) Expression pattern of hnRNPA1 at different stages of preimplantation mouse embryos analyzed using qPCR. 1 C, 1-cell stage; 2 C, 2-cell stage; 4 C, 4-cell stage; 8 C, 8-cell stage; MO, morula; BL, blastocyst. Approximately 50 embryos of each stage were used, and n = 3 biologically independent experiments (**D**) Immunofluorescence staining of hnRNPA1 in embryos at different developmental stages. 1-cell (n = 8), 2-cell (n = 11), 4-cell (n = 7), 8-cell (n = 9), morula (n = 9), blastocyst (n = 10). Scale bar: 50 μm (**E**) Fluorescence intensity analysis showed that hnRNPA1 was highest expressed at 2-cell stage embryos. 1-cell (*n* = 8), 2-cell (*n* = 11), 4-cell (*n* = 7), 8-cell (*n* = 9), morula (*n* = 9), blastocyst (*n* = 10). *n* = 3 biologically independent experiments (**F**) Co-IP results in 2-cell stage embryos (*TUG*1-MS2 was injected at the 1-cell stage) using anti-HA (for HA-labeled MS2 coat protein). The results show that *TUG1* forms an RNA-protein complex with HNRNPA1. TUG1-Rev, the reverse 1500‑bp sequence of *TUG1*; *TUG1*, *TUG1* overexpression; The primer of RT-PCR *TUG1* were used to test *TUG1*; For each co-IP assay, about 800 2-cell embryos were used, and *n* = 3 biologically independent experiments

It is well known that lncRNAs often function through interactions with proteins. Therefore, to elucidate the mechanism of *TUG1* in embryonic development, we performed an RNA pull-down assay using 6,000 two-cell stage embryos to identify proteins interacting with *TUG1*. Using biotin-labeled full-length *TUG1* as the experimental group and a 1500-bp reverse *TUG1* (*TUG1*-Rev) sequence as the negative control, we identified a specific band corresponding to Heterogeneous nuclear ribonucleoprotein A1 (hnRNPA1) by mass spectrometry analysis (Fig. 4A). The interaction between *TUG1* and hnRNPA1 was further confirmed by western blotting (Fig. 4B). qPCR and immunofluorescence (IF) results showed that hnRNPA1 is expressed at relatively high levels during the two-cell stage (Fig. 4C and E). Interestingly, we observed hnRNPA1 signals in the cytoplasm from the one-cell to four-cell stages. In blastocysts, hnRNPA1 was localized only in the TE, with almost no signal detected in the ICM (Fig. 4D). To verify the existence of the *TUG1*-hnRNPA1 complex, we co-injected HA-tagged MS2 coat protein and MS2-tagged *TUG1* at the one-cell stage to achieve overexpression, while co-injection of MS2-tagged reverse *TUG1* with HA-tagged MS2 coat protein served as the negative control. Co-immunoprecipitation (co-IP) was then performed at the two-cell stage. Western blot results confirmed that *TUG1* indeed forms an RNA-protein complex with hnRNPA1 (Fig. 4F).

### The key binding sequences between *TUG1* and hnRNPA1

**Fig. 5:**
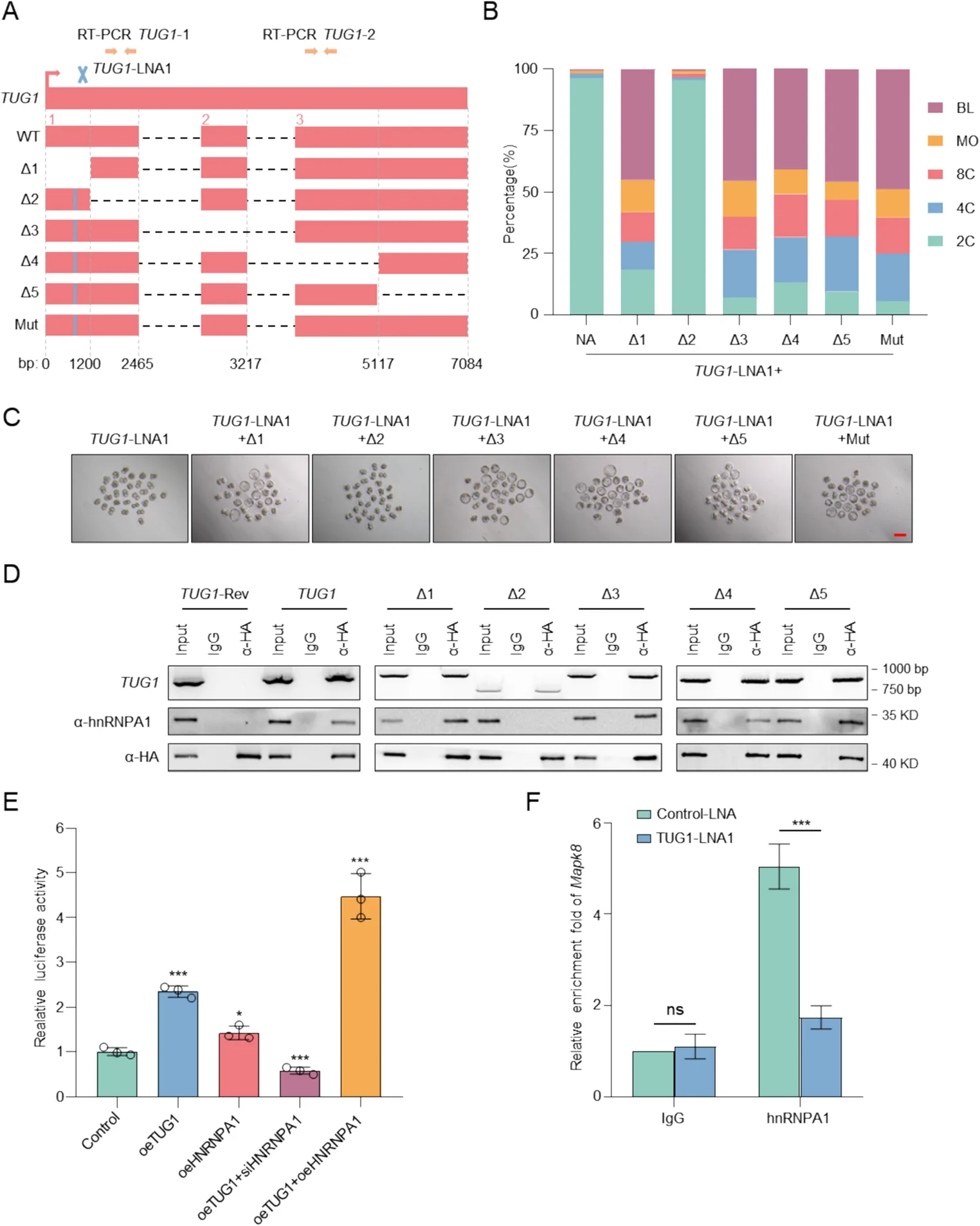
*TUG1* forms a complex with hnRNPA1 to regulate MAPK8 transcription (**A**) Experimental scheme to analyze the truncated sequence of *TUG1*. Mut, *TUG1* with mutation of *TUG1*-LNA1 site. WT, wild type; RT-PCR *TUG1*-1, -2; The RT-PCR primer of *TUG1 * (**B**) Statistical analysis of developmental rates following co-injection of *TUG1*-LNA1 and *TUG1* truncated sequences for overexpression at the 1-cell stage. The bar graph illustrates the developmental rates of each group at the blastocyst stage. *n* = 3 biologically independent experiments (**C**) Embryonic morphology following co-injection of *TUG1*-LNA1 and *TUG1* truncated sequences for overexpression at the 1-cell stage. Scale bar: 100 μm (**D**) Co-immunoprecipitation (Co-IP) in 293T cells using anti-HA antibody (targeting HA-tagged MS2 coat protein) confirms *TUG1*-hnRNPA1 complex formation. *TUG1*-Rev: the reverse 1500‑bp sequence of *TUG1*; α-: anti-. Assays used ≈ 1 × 10^6^ cells per replicate. *n* = 3 biologically independent experiments (**E**) The luciferase reporter assay showed that overexpression of either *TUG1* or hnRNPA1 in 293T cells enhanced the luciferase activity driven by the MAPK8 promoter (F) CUT&Tag-qPCR analysis revealed significant enrichment of hnRNPA1 at the MAPK8 promoter in control embryos, whereas only background signals were detected in IgG controls

In view of the high expression of hnRNPA1 at the 2‑cell stage and its interaction with *TUG1*, we further investigated the role of hnRNPA1 in early embryonic development. Injection of hnRNPA1‑specific interfering fragments at the zygotic stage resulted in developmental arrest at the 2‑ to 4‑cell stage, accompanied by a marked decrease in the expression of ZGA‑associated genes. (Supplementary Fig. S4A-4 C) These findings recapitulate the phenotype induced by *TUG1* depletion. To investigate the key sequences involved in the regulatory mechanism of *TUG1*, we generated five truncated mutants of *TUG1* (Δ1 to Δ5) based on its exon distribution. To avoid interference from the *TUG1*-LNA targeting site, we introduced mutations into the LNA target region within each truncated mutant. Additionally, a full-length *TUG1* construct with mutations in the LNA target site was used as a mutated control (Fig. 5A). Embryos were divided into seven groups and these truncated fragments were overexpressed in embryos subjected to *TUG1* knockdown. Developmental data revealed that injection of Δ1, Δ3, Δ4, Δ5, and the Mut control, but not Δ2 or the reverse fragment (Rev), could rescue the developmental arrest caused by *TUG1* depletion (Fig. 5B, C).

To further explore the specific regulatory mechanism, we employed the MCP-MS2 (MS2 bacteriophage coat protein) system for experiments in 293T cells. The results showed that Δ1, Δ3, Δ4, and Δ5 were able to bind to hnRNPA1, whereas Δ2 could not bind to hnRNPA1 (Fig. 5D). Subsequently, we performed co-immunoprecipitation (co-IP) followed by RT-PCR targeting *TUG1* for each group, successfully detecting the presence of *TUG1* (Fig. 5D). This indicates that *TUG1* exerts its regulatory function by binding to hnRNPA1 through a region spanning nucleotides 1200–2465 within its first exon.

### *TUG1* recruits hnRNPA1 to the MAPK8 promoter

The hnRNP family plays multiple roles in transcriptional and post-transcriptional regulation, serving as major splicing factors and transcriptional regulators [33, 34]. We hypothesized that the RNA-protein complex formed by *TUG1* and hnRNPA1 promotes the expression of MAPK8. To test this, we first assessed the expression of *Mapk8* in hnRNPA1-deficient embryos and found that *Mapk8* transcription was markedly decreased upon hnRNPA1 interference (Supplementary Fig. S4D). In addition, in *TUG1*-deficient embryos, all truncation mutants except hnRNPA1-Δ2 were able to rescue MAPK8 expression, albeit to different extents (Supplementary Fig. S4E). Subsequently, we conducted a dual-luciferase reporter assay in 293T cells. The results showed that overexpression of *TUG1* alone (approximately 2.3-fold) or hnRNPA1 alone (approximately 1.4-fold) enhanced the luciferase activity of the MAPK8 promoter, indicating that hnRNPA1 itself acts as a positive regulator of MAPK8 (Fig. 5E). Moreover, overexpression of *TUG1* alone resulted in stronger luciferase activity (approximately 2.3-fold), suggesting that *TUG1* may recruit additional hnRNPA1 to the MAPK8 promoter (Fig. 5E). Furthermore, when *TUG1* and hnRNPA1 were co-overexpressed, the luciferase activity of the MAPK8 promoter was greatly enhanced (approximately 4.5-fold), indicating a synergistic rather than simply additive functional interaction between the two (Fig. 5E). To determine whether hnRNPA1 directly associates with the MAPK8 regulatory region in early embryos, we performed CUT&Tag-qPCR using an anti-hnRNPA1 antibody in control-LNA and *TUG1*-LNA1 two-cell embryos. CUT&Tag-qPCR analysis showed robust enrichment of hnRNPA1 at the MAPK8 promoter in control embryos compared with IgG controls, indicating specific occupancy of hnRNPA1 at this locus (Fig. 5F). Importantly, *TUG1* knockdown significantly reduced hnRNPA1 enrichment at the MAPK8 promoter, whereas no enrichment was detected in the negative control regions. These findings suggest that *TUG1* is required for efficient recruitment or stabilization of hnRNPA1 at the MAPK8 promoter, thereby supporting MAPK8 transcription during zygotic genome activation.

**Fig. 6:**
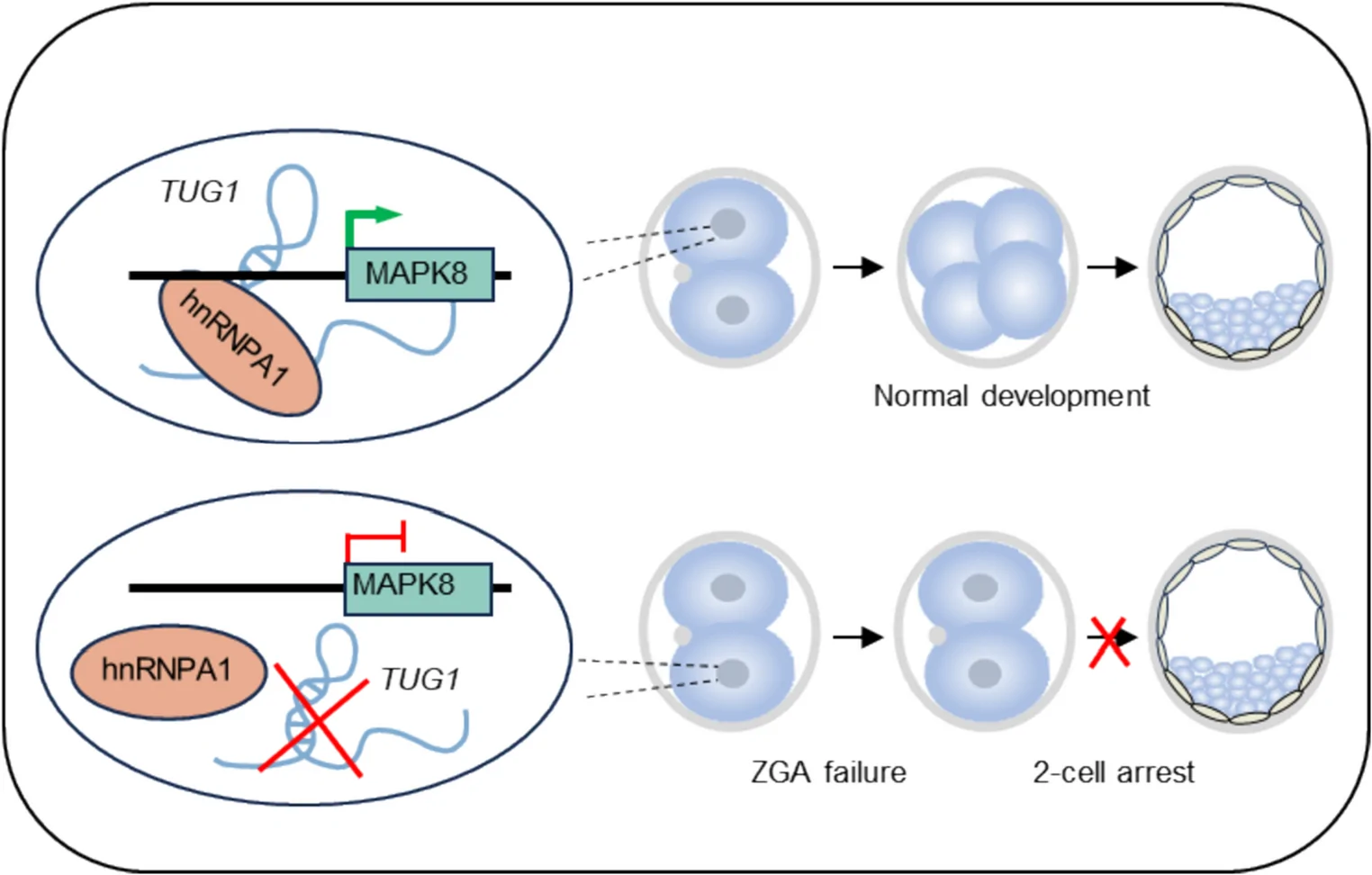
Model showing that *TUG1* regulates MAPK8 by binding to hnRNPA1

Therefore, our findings demonstrate that *TUG1* can interact with hnRNPA1 through the nucleotide region 1200–2465 within its exon to form an RNA-protein complex, which drives the MAPK signaling pathway by promoting the transcription of MAPK8, playing a crucial role in early mouse embryonic development (Fig. 6).

## Discussion

In this study, we investigated the underlying mechanisms through which the pro-oncogenic lncRNA *TUG1* regulates early mouse embryonic development. We found that *TUG1* is highly expressed at the two-cell stage and its depletion leads to embryonic arrest at this stage, indicating its critical role in early mouse embryogenesis. RNA-Seq analysis revealed that *TUG1* deficiency affects the MAPK signaling pathway, with its key factor MAPK8 being downregulated. Mechanistically, we discovered that *TUG1* directly interacts with hnRNPA1 and associates with the MAPK8 promoter. Through binding to hnRNPA1, *TUG1* promotes MAPK8 transcription and the onset of ZGA.

Recent research has highlighted the crucial roles of lncRNAs during early embryonic development. GLN-, MERVL-, and ERVK-associated lincRNAs, such as LincGET, participate in alternative splicing during major ZGA in mouse embryos [35]. The SINE-associated lncRNA SAWPA regulates porcine ZGA via the JNK-MAPK signaling pathway [13]. The enhancer-like lncRNA FKBPL modulates porcine zygotic gene expression by interacting with HSP90AA1 and CDK9 [14]. Among cancer-associated lncRNAs, *TUG1* has emerged as a prominent and actively studied player. *TUG1* is highly expressed in various cancer types and promotes cancer cell proliferation and invasion through diverse regulatory mechanisms [36–38]. However, its function in early embryonic development has remained largely unexplored. In this study, we report for the first time the expression profile and functional mechanism of *TUG1* in early mouse embryos.

Furthermore, we identified a distinctive localization pattern of *TUG1* at the one-cell stage. RNA-FISH results showed that *TUG1* localizes to the subcortical region in one-cell embryos and partially translocates into the nucleus by the two-cell stage. The subcortical maternal complex (SCMC), a group of maternally derived proteins localized beneath the cortex of oocytes and one-cell embryos, plays a key role during the oocyte-to-embryo transition (OET) [39, 40]. It has been demonstrated that the SCMC is composed of multiple maternal proteins, including MATER, TLE6, FLOPED, FILIA, ZBED3, NLRP4F, NLRP2, NLRP9, NLRP14, PADI6, and SPIN1 [39–43]. Depletion of these proteins leads to embryonic arrest at the two-cell stage. Our mass spectrometry data also identified several SCMC components that interact with *TUG1*. Whether the interaction between *TUG1* and these SCMC proteins influences the (OET) transition will be a focus of our future investigations.

Although the interaction between *TUG1* and hnRNPA1 was supported by RNA pull-down and Co-IP analyses, additional biochemical controls, including competition assays and RNase-dependent validation, would further strengthen the specificity of this interaction and warrant future investigation. Additionally, we observed another interesting phenomenon, hnRNPA1 localizes specifically to the TE in blastocysts. hnRNPA1 is a ubiquitously expressed RNA-binding protein of the HNRNP family, involved in diverse cellular functions such as transcription factor regulation, alternative splicing, mRNA stability, mRNA nuclear export, and miRNA processing [34, 44–46]. However, the function of hnRNPA1 in early embryonic development remains largely unexplored. We speculate that its polarized distribution at the blastocyst stage may participate in HIPPO-signaling-mediated lineage segregation, which will be investigated in our future work.

The identification of a *TUG1*–hnRNPA1–MAPK8 signaling axis in early embryos also raises intriguing questions regarding the evolutionary conservation of *TUG1* function across physiological and pathological settings. Given the well-established roles of *TUG1* in multiple human malignancies and the frequent dysregulation of MAPK signaling in cancer, it is tempting to speculate that developmental regulatory modules involving *TUG1* may be reutilized in tumor cells. Future investigations examining whether *TUG1* controls MAPK8 expression through analogous mechanisms in cancer systems may provide important insights into the shared molecular principles governing embryonic development and tumor progression.

## Supplementary Information

Below is the link to the electronic supplementary material.

Supplementary Material 1 (DOCX 568 KB)

Supplementary Material 2 (XLSX 12.6 KB)

## Data Availability

Publicly available clinical cohorts utilized in this study were sourced from: The Cancer. Genome Atlas (TCGA; https://www.cancer.gov/tcga), Gene Expression Omnibus (GEO; https://www.ncbi.nlm.nih.gov/geo/) with the accession number of PRJCA067358. The authors declare that the data supporting the findings of this study are available within the article and its Supplementary Information files. All relevant data are available from the authors upon request.
