# Supplementary material for: TUG1 supports mouse two-cell-stage development and ZGA-associated transcription through MAPK8 signaling
Source: Cell Mol Life Sci. 2026 Aug 11;83(1):412. doi: 10.1007/s00018-026-06374-z (PMC13748198; doi:10.1007/s00018-026-06374-z)
Supplement: Supplementary file 1 [file 18_2026_6374_MOESM1_ESM.docx]

**Supplementary Figures**

**Supplementary Figure S1. Expression profiles of candidate lncRNAs in mouse preimplantation embryos**

**(A-G)** Line plots showing the lncRNA profiles of *TUG1*, *MALAT1*, *SNHG1*, *ANHG3*, *SNHG6*, *SNHG9* and *GAS5* in various developmental stages of mouse preimplantation embryos were examined by RNA-seq (Single-cell RNA-seq data source: GSE45719). Data are presented as mean values ± SEM.

**Supplementary Figure S2. TUG1 knockdown arrests embryonic development at the two‑cell stage without impairing DNA replication**

**(A)** Statistical analysis of the effect of *TUG1*-LNA injection on embryonic development rates. Data represent mean ± SEM from at least 3 experimental replicates. Statistical significance was determined by Student's t-test. *P < 0.05; **P < 0.01; ***P < 0.001.

**(B)** *TUG1* depletion results in developmental arrest at the two-cell stage without affecting DNA integrity and replication. Aphidicolin-treated embryos arrest at the S phase without DNA replication. LNA was injected at phCG 25 h, and embryos were collected at phCG 48 h at the late two-cell stage for IF analysis. Scale bar, 50 μm. *n* = 3 biologically independent experiments.

**Supplementary Figure S3. RNA‑seq analysis of 2‑cell‑arrested embryos upon *TUG1* knockout**

**(A)** Heatmap of differentially expressed gene profiles in zygotes injected with control‑LNA or TUG1‑LNA. Two replicates were performed per group.

**(B)** Pie chart shows the percentage of downregulated genes that overlap with major ZGA, minor ZGA, and maternal genes in late 2‑cell embryos injected with TUG1‑LNA.

**Supplementary Figure S4. Depletion of hnRNPA1 results in 2‑cell embryonic arrest and downregulation of *Mapk8* expression.**

**(A)** Embryonic morphology at preimplantation stages following hnRNPA1 knockdown. Scale bar: 100 μm.

**(B)** Embryonic development after microinjection. Different letters in same column indicate significant difference (*p* < 0.001).

**(C)** hnRNPA1 knockdown reduces expression of ZGA-related genes. Embryos injected with siRNA were collected at phCG 48 h at the late two-cell stage for qPCR analysis. The error bars represent SEM. About 50 embryos were used, and three experimental replicates were performed. For all panels, error bars represent SEM. Significance was determined by two-tailed t-test, **p* ≤ 0.05; ***p* ≤ 0.01; ****p* ≤ 0.001; *****p* ≤ 0.0001.

**(D)** hnRNPA1 knockdown reduces expression of *Mapk8*. Embryos injected with siRNA were collected at phCG 48 h at the late two-cell stage for qPCR analysis. The error bars represent SEM. About 50 embryos were used, and three experimental replicates were performed. For all panels, error bars represent SEM. Significance was determined by two-tailed t-test, **p* ≤ 0.05; ***p* ≤ 0.01; ****p* ≤ 0.001; *****p* ≤ 0.0001.

**(E)** Detection of MAPK8 expression changes following injection of TUG1 LNA1 and overexpression of TUG1 truncated constructs at the 1‑cell stage. The error bars represent SEM. About 50 embryos were used, and three experimental replicates were performed. For all panels, error bars represent SEM. Significance was determined by two-tailed t-test, **p* ≤ 0.05; ***p* ≤ 0.01; ****p* ≤ 0.001; *****p* ≤ 0.0001.

**Supplementary Materials**

**BrdU Staining**

Embryos were fixed with 4% PFA (Paraformaldehyde) for 30 minutes, washed twice with PBS, and then transferred to 1.5 M hydrochloric acid solution and incubated at room temperature for 30 minutes. After two additional washes with PBS, the embryos were placed in 500 µL of permeabilization solution and incubated overnight (≥10 h) at 4 °C. Following two PBS washes, the embryos were blocked for 1 hour and then incubated with primary antibody overnight (≥10 h). After two PBS washes, fluorescently labeled secondary antibody was added and incubated at room temperature for 2 hours in the dark. The embryos were washed twice with PBS and then stained with DAPI for nuclear labeling, incubated in the dark for 10 minutes, and washed twice with PBS. Finally, the processed embryos were mounted on glass slides, covered with coverslips using mounting medium, and imaged using a confocal microscope.

**EU Staining**

EU labeling was performed using the Click‑iT® RNA Alexa Fluor™ 488 Imaging Kit. Embryos were incubated in KSOM medium containing 10 μM Component A for 3 h at 37 °C in a 5% CO₂ incubator. Subsequently, the embryos were immersed in 4% paraformaldehyde fixative and incubated at room temperature for 30 minutes in the dark. After two washes with PBS, the embryos were transferred to permeabilization solution and incubated at room temperature for 15 minutes, followed by a 5‑minute wash with PBS. The Click‑iT® reaction cocktail was prepared by combining 428 μL of 1×Component C, 20 μL of Component D, 2 μL of Component B, and 50 μL of 1×Component E. Embryos were then transferred into the Click‑iT® reaction cocktail and incubated at room temperature in the dark for 30 minutes. After washing with Component F for 5 minutes, the embryos were rinsed three times with PBS (5 minutes each). Nuclear staining was performed by incubating with DAPI in the dark for 10 minutes, followed by two washes with PBS. Finally, embryos were mounted on glass slides, covered with coverslips using an anti‑fade mounting medium, and images were acquired and saved using a confocal microscope.

**RNA Extraction, Reverse Transcription, and In Vitro Transcription**

Total RNA was isolated using TRIzol reagent, followed by DNase treatment with the RNase-Free DNase Set (QIAGEN, #79254) to eliminate genomic DNA contamination. Reverse transcription was subsequently performed using RevertAid Reverse Transcriptase (Thermo Fisher, #EP0451). For in vitro mRNA synthesis, the HiScribe™ T7 ARCA mRNA Kit (NEB, #E2060S) was employed: A 20 μL capped transcription reaction mixture was assembled and incubated at 37 °C for 30 min, followed by template DNA degradation throUGh DNase I treatment (2 μL, 37 °C, 15 min). Polyadenylation was then conducted at 37 °C for 45 min, with final RNA purification achieved via 25 μL lithium chloride precipitation.

**Quantitative Real-Time PCR analysis**

Total RNA was extracted from collected embryos and reverse-transcribed into cDNA, which was stored at -20°C until analysis. Gene expression quantification was performed using TB Green Premix Ex Taq (Takara Bio, RR420A) on a real-time PCR system under the following thermal profile: initial denaturation at 95 °C for 30 s, 40 cycles of denaturation (95 °C, 5 s) and annealing/extension (60 °C, 34 s), followed by a dissociation stage (95 °C for 15 s and 60 °C for 60 s). All reactions were conducted in triplicate, with target gene expression normalized to reference genes using the 2^(-ΔΔCt) method for relative quantification.

**RNA-Seq and Analysis**

For each biological replicate, 1000 two-cell embryos were collected, and two independent biological replicates were generated for both the control and TUG1 knockdown groups. Total RNA was extracted using the miRNeasy Mini Kit (Qiagen, #217004), where samples were lysed with QIAzol and purified via spin columns to obtain high-quality RNA. Subsequently, the Trance RNAseq Library Prep Kit (KAITAI-Bio, #AT4205) was employed for reverse transcription, synthesizing cDNA through Oligo(dT) primers and template switching, followed by pre-amplification. The cDNA was then purified using DNA size selection beads. During library construction, the cDNA was fragmented and simultaneously ligated with adapters using Tn5 transposase, followed by index introduction via PCR. Finally, two rounds of bead-based size selection were performed to precisely isolate library fragments ranging from 400 bp to 600 bp. The quality of the library was verified by PAGE electrophoresis and Qubit

quantification, ensuring a final product with a focused size distribution and a concentration exceeding 1 ng/μL. Qualified libraries were sequenced on the Illumina

platform using a paired-end 150 bp strategy.

Raw sequencing data were first processed and filtered using fastp (v0.23.4) to obtain high-quality Clean Data; the Clean Data were then aligned to the reference genome using HISAT2 (v2.2.1), and the resulting alignments were converted to BAM files using SAMtools (v1.16.1), with alignment quality assessed by RSeQC (v5.0.1); transcript assembly was performed using StringTie (v2.2.1), and novel transcripts were identified through comparison with known annotations using gffcompare (v0.12.6); gene expression quantification was carried out with HTSeq (v0.13.5) to generate read counts, which were normalized using FPKM, followed by principal component analysis and sample correlation analysis based on the expression matrix; differential expression analysis was conducted using the edgeR package (v3.36.0) with screening criteria set at |log2(fold change)| ≥ 1 and adjusted p-value ≤ 0.05; the identified differentially expressed genes were subsequently subjected to GO, KEGG, and Reactome enrichment analyses using the clusterProfiler package (v4.2.0).

**Dual-Luciferase reporter assay**

293T cells were co-transfected with the following constructs: a reporter plasmid encoding firefly luciferase, the pRL-TK plasmid expressing *Renilla* luciferase as a transfection control, and additional expression vectors specified in the results section. After 48 hours of incubation (with cell confluence maintained below 95%), the culture medium was aspirated. Cells were then washed with PBS under gentle agitation to remove non-adherent cells and residual medium. This PBS washing step was repeated twice. Fluorescence intensity was quantified using the Dual-Luciferase Reporter Assay System (Promega, E1910).

**RNA Pull-Down assay**

Utilizing the Pierce™ Magnetic RNA-Protein Pull-Down Kit (Thermo Fisher, #20164), streptavidin magnetic beads were pre-washed twice before incubation with biotinylated target RNA and cell lysates. After 30-minute room-temperature binding with intermittent pipette mixing, the complexes underwent magnetic separation followed by two consecutive washes with 20 mM Tris buffer A, 100 μL Protein-RNA binding mixture-comprising 10 μL Binding Buffer, 30 μL 50% glycerol, 30 μL additional salts, 30 μL lysat and nuclease-free water—was added to the RNA-bound beads and incubated at 4 °C for 60 min. Post-incubation magnetic separation preserved the supernatant, after which beads were washed three times with Wash Buffer. Target complexes were eluted in 50 μL Elution Buffer via vortex mixing, incubated at 37°C for 30 min, and the final supernatant collected for analysis.

**Western blot analysis**

Protein samples obtained from RNA pull-down assays or extracted from embryos using Pierce™ IP Lysis Buffer (Thermo Fisher, #87787) were mixed with sample buffer and stored at -80°C. For analysis, 20 μL aliquots were thawed on ice, combined with 5 μL SDS-PAGE Loading Buffer, and denatured at 100°C for 5 minutes. Following separation gel preparation and addition of electrophoresis buffer, samples (10 μL/lane) were resolved at constant 110 V for 60 minutes. Post-electrophoresis, gels were trimmed and rinsed with deionized water. Proteins were transferred to PVDF membranes using a semi-dry transfer system at 350 mA for 60 minutes with six filter papers and PVDF membranes assembled in transfer cassettes. Membranes were blocked with 3% BSA for 1 hour at room temperature, then incubated with primary antibodies at 4°C overnight. After three 5-minute TBST washes, membranes were treated with secondary antibodies for 1 hour at room temperature, followed by another series of TBST washes. Target proteins were detected by chemiluminescence after 1-minute substrate incubation (ECL reagent) and imaged using a chemiluminescence detection system.

**Immunofluorescence staining**

Embryos at various developmental stages were washed three times in PBS, treated with acidic Tyrode's solution to remove the zona pellucida, and rewashed in PBS. Samples were fixed in 4% PFA for 30 min at room temperature, permeabilized in 1% Triton X-100 for 30 min, and blocked with 3% BSA for 1 hour. Primary antibody incubation was performed overnight at 4°C, followed by five 5-minute washes in antibody wash buffer. Secondary antibodies were applied for 1 h, succeeded by another five 5-minute washes. Embryos were mounted in antifade medium containing 5 μL nuclear counterstain (Thermo Fisher, P36971) and stored at 4°C protected from light.

**Co-immunoprecipitation (Co-IP) assay**

293T cells were co-transfected with MS2P-HA and TUG1-24×MS2 vectors using Lipofectamine LTX & PLUS Reagent (Thermo Fisher, #15338100). For Co-IP, the Pierce Crosslink Magnetic IP/Co-IP Kit (Thermo Fisher, #88805) with HA antibodies crosslinked to Protein A/G Magnetic Beads was employed. Transfected cells were lysed in 100 μL IP Lysis Buffer, with 10 μL lysate reserved as input control. The remaining lysate underwent overnight incubation at 4°C with anti-HA-conjUGated beads. After two 5-minute washes with IP Lysis Buffer, beads were resuspended in 500 μL ultrapure water. Target complexes were eluted in 100 μL Elution Buffer, mixed with 15 μL 6 × SDS loading buffer, and heat-denatured at 100 °C for 5 min. Co-precipitated HNRNPA1 and HA-tagged proteins were detected by western blot.

**CUT&Tag-qPCR**

CUT&Tag-qPCR was performed using the Hyperactive Universal CUT&Tag Assay Kit (Vazyme, #TD904) according to the manufacturer’s instructions. Briefly, a defined number of cells (with H3K4me3 as a positive control and IgG as a negative control) were collected, bound to activated ConA beads, and sequentially incubated with the primary antibody against the target protein (overnight at 4°C) and the corresponding secondary antibody (30–60 min at room temperature). The beads were then incubated with pA/G-Tnp Pro (final concentration 0.04 μM) for 1 h at room temperature, washed, and subjected to tagmentation with the fragmentation buffer at 37°C for 60 min. After stopping the reaction, DNA Spike‑in (1 pg per 100,000 cells) was added as an internal reference, and the fragmented DNA was purified using magnetic beads. The purified DNA was released by heating in Stop Buffer at 95°C for 5 min, and the eluate was used for SYBR Green qPCR amplification (95°C for 30 s, followed by 40 cycles of 95°C for 10 s and 60°C for 30 s). Using DNA Spike‑in as the reference, the ΔCt values for each target gene were calculated as Ct(target) − Ct(Spike‑in), and ΔΔCt was then calculated relative to the IgG negative control. Relative enrichment folds were determined using the 2⁻ΔΔCt method. All samples were run in technical replicates to ensure data reliability.
